# Marginal and Internal Fit of Cobalt-Chromium Fixed Dental Prostheses Generated from Digital and Conventional Impressions

**DOI:** 10.1155/2014/534382

**Published:** 2014-03-03

**Authors:** Per Svanborg, Henrik Skjerven, Pablo Carlsson, Alf Eliasson, Stig Karlsson, Anders Örtorp

**Affiliations:** ^1^Department of Prosthetic Dentistry/Dental Materials Science, Institute of Odontology, Sahlgrenska Academy, University of Gothenburg, Medicinaregatan 12 F, P.O. Box 450, 40530 Gothenburg, Sweden; ^2^Department of Prosthetic Dentistry, University of Oslo, 0161 Oslo, Norway; ^3^Department of Prosthetic Dentistry, Postgraduate Dental Education Center, P.O. Box 1126, 70111 Örebro, Sweden; ^4^School of Health and Medical Sciences, Örebro University, 70281 Örebro, Sweden

## Abstract

*Objectives*. Digital impressions are increasingly used and have the potential to avoid the problem of inaccurate impressions. Only a few studies to verify the accuracy of digital impressions have been performed. The purpose of this study was to compare the marginal and internal fit of 3-unit tooth supported fixed dental prostheses (FDPs) fabricated from digital and conventional impressions. *Methods*. Ten FDPs were produced from digital impressions using the iTero system and 10 FDPs were produced using vinyl polysiloxane (VPS) impression material. A triple-scan protocol and CAD software were used for measuring and calculating discrepancies of the FDPs at 3 standard areas: mean internal discrepancy, absolute marginal gap, and cervical area discrepancy. The Mann-Whitney U test was used for analyzing the results. *Results*. For conventional and digital impressions, respectively, FDPs had an absolute marginal gap of 147 **μ**m and 142 **μ**m, cervical area discrepancy of 69 **μ**m and 44 **μ**m, and mean internal discrepancy of 117 **μ**m and 93 **μ**m. The differences were statistically significant in the cervical and internal areas (*P* < 0.001). *Significance*. The results indicated that the digital impression technique is more exact and can generate 3-unit FDPs with a significantly closer fit compared to the VPS technique.

## 1. Introduction

The lost wax technique is being rapidly replaced by computer-aided design/computer-aided manufacturing (CAD/CAM) techniques in dentistry and dental technology [1], and the quality and fit of these restorations need to be evaluated. An important factor in determining clinical longevity is the fit of the restoration, and both marginal fit and internal fit have to be considered [2, 3]. A mean marginal gap of 100 *μ*m has been regarded as clinically acceptable [4–7].

Traditional techniques for measuring fit have limitations, not only in being restricted to 2D images, but also in suffering from a limited number of measuring points [3]. A new technique for 3D fit evaluation has been proposed by Holst et al. [8], using a triple scan protocol to obtain detailed information of component precision in all spatial orientations. Furthermore, the technique allows specific areas to be evaluated and the absolute marginal gap to be measured. However, this is not the same as the mean marginal gap since it includes the under- and/or overextension of the crown as well as the marginal gap [2].

Titanium single crowns produced with conventional impressions and CAD/CAM technology have demonstrated mean marginal gaps in the range of 18–88 *μ*m [9–12]. Cobalt-Chromium (CoCr) crowns fabricated with the direct laser metal sintering technology (DLMS) demonstrated a mean marginal gap of 93 *μ*m [13].

The internal fit of crowns has not been studied to the same extent as the marginal fit, despite its importance for the retention of restorations. Internal fit can be divided into axial and occlusal areas, and for titanium crowns, internal discrepancies in the range of 93–127 *μ*m for axial area and 161–177 *μ*m occlusal area have been reported [10]. CoCr crowns made using the DLMS technique have demonstrated occlusal discrepancies in the range of 252–284 *μ*m [13]. Ucar and colleagues evaluated fit of cast and DLMS fabricated CoCr crowns, reporting mean internal discrepancies (occlusal and axial) of 51–63 *μ*m [14]. The fit of 3-unit FDPs fabricated in a CoCr alloy using different production techniques has been evaluated, and a mean internal discrepancy of 84 *μ*m was reported for the DLMS technique and 166 *μ*m for the milling technique [3]. For both groups, the discrepancies were larger at the margin and in the occlusal areas compared to the axial areas.

Several intraoral scanning devices are now available on the market. The potential advantages of digital impressions and a digital workflow are the elimination of production steps that may cause misfit, less transport between clinic and dental laboratory, and less patient discomfort [15].

A few studies have compared the fit of single crowns fabricated with digital and conventional impression techniques, reporting comparable results for both techniques [16–19]. Studies on multiunit implant supported FDPs or dental arches comparing CAD files also show comparable results [15, 20, 21]. Almeida et al. [22] compared the fit of four-unit zirconia FDPs generated from digital and conventional impressions. Using the replica method, the digital impressions (3 M LAVA COS) demonstrated a mean internal fit of 58 *μ*m and the conventional impressions 66 *μ*m. The marginal gaps were 64 *μ*m and 65 *μ*m, respectively. However, no studies have been published concerning the fit of tooth-supported multiunit CoCr FDPs produced from digital impressions.

The aim of this study is to evaluate the marginal and internal fit of CNC-milled CoCr 3-unit FDPs produced from digital and conventional impressions using a triple-scan protocol for 3D fit assessment. The null hypothesis is that there is no difference in fit of FDPs produced from digital and conventional impression techniques. The alternative hypothesis is that there is a difference in fit of the FDPs, and the direction of the difference is yet unknown.

## 2. Materials and Methods

### 2.1. Pilot Study

A pilot study was performed to determine the design of the master cast, to ensure that the optical measuring technique was appropriate for the chosen design.

One acrylic master cast was fabricated with premolar tooth 34 and molar tooth 36 prepared for a 3-unit FDP. The preparations were circumferent: 360° deep chamfer, a preparation depth of 1 mm for the axial area and 2 mm for the occlusal areas, and a convergence angle of 10°. The master cast was duplicated using type IV stone (Shera Hard Rock, Shera Werkstoff Technologie GmbH & Co., Lenförde, Germany), which is recommended for scanning. A conventional impression was made using an individually designed impression tray (Photo-Tray, light-curing baseplates, Dentalfarm, Torino, Italy) and vinyl polysiloxane (VPS) impression material (Honigum, DMG, Hamburg, Germany). The impression was cast with a type IV stone (Shera Hard Rock). A CoCr 3-unit FDP (Heraenium PW, Heraeus Kulzer GmbH, Hanau, Germany), fabricated using the lost wax technique on a working cast (Induction/vacuum pressure casting machine, Heracast iQ, Heraeus Kulzer), was placed on the original stone cast. The placement of the abutment teeth, finishing line, and the position of the neighboring teeth (33 and 37) were evaluated after scanning with both the digital impression technique and the ATOS III triple-scan (GOM mbH, Braunschweig, Germany) scanner used for analysis of fit. The original stone cast was also analyzed together with the FDP. A slight change in angles 33 and 37 was performed to ensure that the ATOS scanner, which uses triangulation, would capture the mesial finish line of 34 and the distal finish line of 36.

### 2.2. Study Casts

To standardize the study casts, the master cast was duplicated using four silicone molds (Zhermack duplication silicone, elite double 32 extra fast, Zhermack SpA, Badia Polesine, Italy). The molds were cast with type IV stone (Shera Hard Rock) to achieve 20 casts. The 20 study casts were assigned by blind randomization to control and test groups, with 10 casts in each group.

### 2.3. Test Group

**Figure 1:**
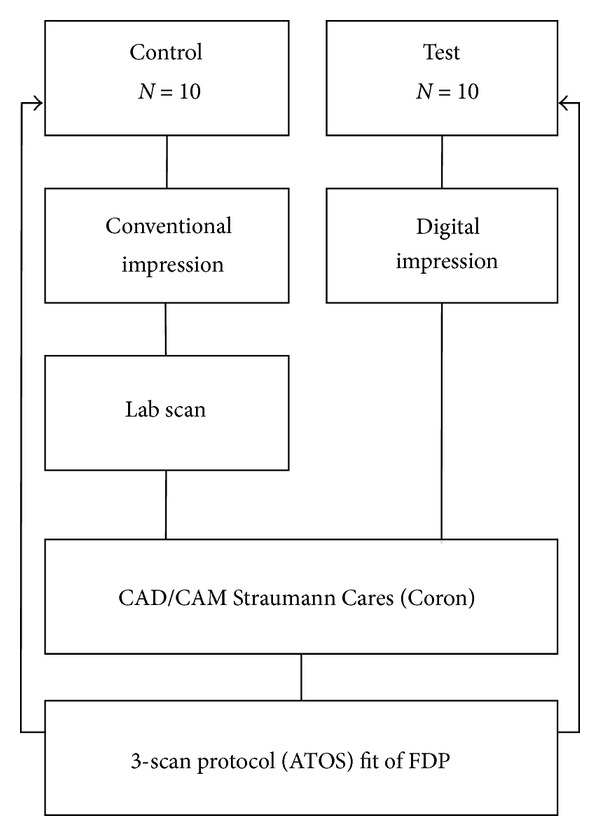
Study design comparing the fit of fixed dental prostheses produced with the conventional and digital impression techniques.

An intraoral scanner (Cadent iTero, Tel Aviv, Israel) was used to perform the digital impressions of the 10 test casts (Study design, Figure 1). The scanner was managed by one person only. The hand-held scanner uses the confocal light technique to capture images. A series of five scans per abutment were taken, followed by additional scans to record the remainder of the quadrant and antagonist. After an assimilation process, a 3-dimensional model of the scanned arch was produced. The scan was controlled and the preparation margin was checked. The file was then sent to a workstation and prepared by one of the authors with reference to preparation margin and insertion path. The files were then downloaded to a Straumann Cares workstation and 3-unit FDPs with a cutback design for metal ceramics were designed using Cares CAD software (CARES VISUAL 6.2). The cement gap (0.5 mm closest to the finish line) was set at 30 *μ*m, the spacer gap (beginning above the cement gap) was set at 60 *μ*m, and the correction of milling radius was set at 110% in the cusp areas. The minimum restoration thickness was set at 0.4 mm, the thickness at the finishing line was set at 80 *μ*m, and the cutting angle was 20°. The CAD files were then transmitted to a production facility (Straumann, Leipzig, Germany) where the frameworks were CNC-milled (Coron Co Balance %, Cr 28%, W 8.5%, Si 1.65%, Mn < 1%, N < 1%, Nb < 1%, Fe < 1%, Straumann). The marking of finishing lines, approval of intraoral scans, and designing of all FDPs were performed by one of the authors.

### 2.4. Control Group

The conventional impression technique was performed by one of the authors using individually designed impression trays (Photo-Tray). An adhesive for VPS impression material (VPS Tray Adhesive, 3 M ESPE, Seefeld, Germany) was applied to the trays 5–10 minutes before impression. Putty-wash impressions were taken using VPS material (Honigum light body and heavy body impression material). Impressions were set under finger pressure for 5 minutes at room temperature. One experienced prosthodontist and one experienced dental technician examined the impressions for tears and voids and connection between tray and impression material. The impressions were poured with type IV stone (Shera Hard Rock). Conventional laboratory procedures were used to fabricate a working cast with removable sections. The abutment sections were ground, using conventional burs for stone, for easier access to the preparation lines. The casts were scanned using a Straumann Cares scanner and the files were transmitted into the Cares CAD software (CARES VISUAL 6.2). Three-unit FDPs were produced using the same settings and the same operator as for the test group.

### 2.5. Framework Check-Up

All FDPs were tested on their respective study cast upon arrival. One FDP from the control group was considered clinically unacceptable due to misfit; that is, it fitted well to the working cast but not to the study cast. The impression technique and/or working cast production were considered the reason for misfit. The FDP was therefore not used and a new impression was made on the study cast. From the new impression, a working cast was made and a new FPD was fabricated and scanned according to the established protocol. The frameworks were not adapted for a better fit but measured in the as-delivered state.

### 2.6. Measuring of Working Casts and Frameworks

A noncontact ATOS III triple-scan (GOM) scanner with blue-light technology was used to determine the fit of the FDPs to the study casts, using triangulation. The scanning was performed according to the triple-scan protocol for 3D fit assessment used by Holst et al. [8]. According to Holst et al., the repeatability of the technique was almost perfect, revealing an intraclass correlation coefficient of *r* = 0.981 [8]. First the cast and the inside of the restoration were scanned separately; then, a positioning scan with the restoration placed on the cast was performed. The distances from the abutment surface to the inside of the FDP were measured from 250,000 to 400,000 points per abutment resulting in a point cloud representing the discrepancy between the two surfaces. Experienced operators performed the scanning procedure. The scanning information was then put together in CAD software (GOM Inspect v7.5, GOM mbH, Braunschweig, Germany), and the discrepancy between each abutment and the corresponding inner surface of the retainer was calculated for the FDP. The internal discrepancy was calculated as a mean of all distances between abutment and inner surface of the retainer. The absolute marginal gap was measured from the finish line to the crown margin circumferential and a mean was obtained using the CAD software, and hence this measurement includes both marginal gap and under- or overextension of the crown. The cervical area discrepancy was measured from the 0.5 mm closest to the finish line circumference. All analyses of discrepancies were performed by two of the authors. The settings for distance measurements were maximum distance 0.4 mm, maximum deviation of normals 60°, and maximum opening angle 30°.

### 2.7. Statistical Analysis

The fit of the FDPs was compared in terms of the impression method in three ways: mean internal discrepancy, absolute marginal gap, and cervical area discrepancy. The Mann-Whitney *U* test was used to detect significant differences between the test and the control group. Normal distribution was confirmed using box plots. The significance level was set at *P* < 0.01.

## 3. Results

The conventional impression technique suffered a major complication for one FDP that had to be remade, since the FDP could not be seated on the corresponding study cast due to serious misfit. All FDPs produced with the digital impression technique were easily seated on their study casts.

The preset cement spacer settings were identical for both techniques, 30 *μ*m in the cervical area, from the finish line and 0.5 mm axially, and 60 *μ*m above the cervical area for the axial and occlusal areas. The mean internal discrepancy for the FDPs was 117 *μ*m and 93 *μ*m, respectively, for the conventional and digital impression techniques; *P* < 0.001 (Table 1).

**Figure 2:**
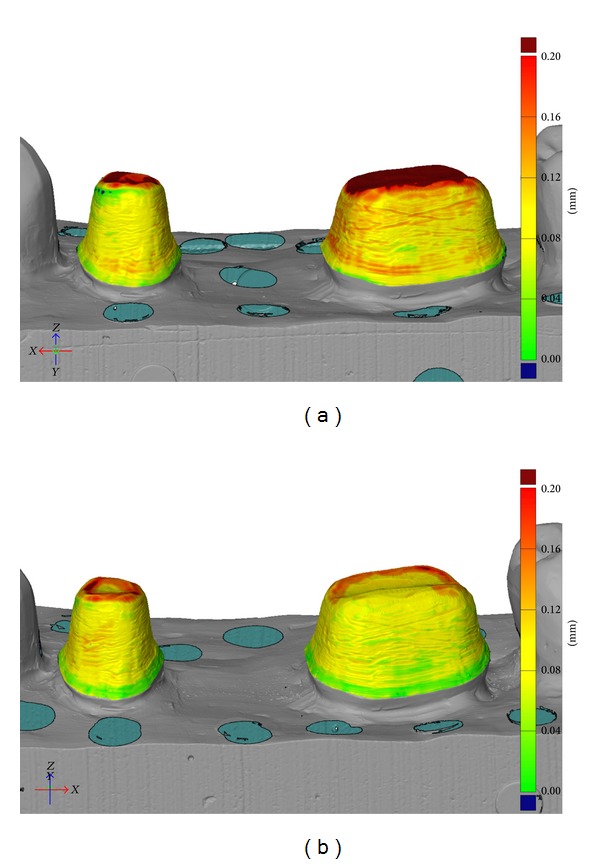
(a) Fit analysis of FDPs produced with conventional impression technique. The distance from abutment to inside of FDP is represented by color. Green = 0–40 *μ*m; yellow = 70–110 *μ*m; red = 160–200 *μ*m. (b) Fit analysis of FDPs produced with digital impression technique. The distance from abutment to inside of FDP is represented by color. Green = 0–40 *μ*m, yellow = 70–110 *μ*m; red = 160–200 *μ*m.

**Figure 3:**
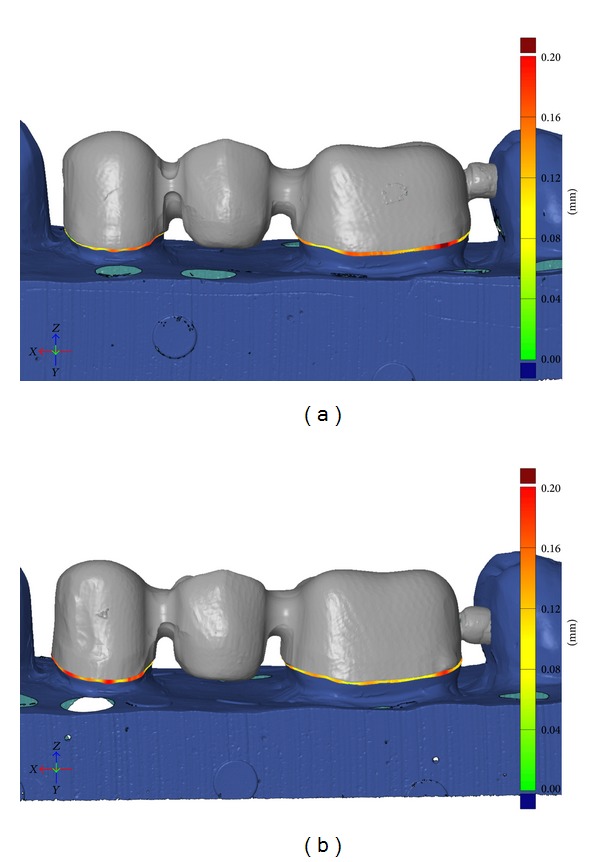
(a) Analysis of the absolute marginal discrepancy of FDPs produced with the conventional impression technique. The distance from finishing line to the margin of restoration is represented in color. Green = 0–40 *μ*m; yellow = 70–110 *μ*m; red = 160–200 *μ*m. (b) Analysis of the absolute marginal discrepancy of FDPs produced with the digital impression technique. The distance from finishing line to the margin of restoration is represented in color. Green = 0–40 *μ*m; yellow = 70–110 *μ*m; red = 160–200 *μ*m.

In general, the distance between the abutment and inner surface of the FDP was the shortest just above the finishing line and the greatest in the occlusal area. The digital impression technique produced FDPs with less discrepancy in the occlusal and cervical area as well as along the axial walls (Figures 2(a) and 2(b)). The mean absolute marginal gap was 147 *μ*m for the conventional technique and 142 *μ*m for the digital impressions, with no statistically significant difference (Table 2, Figures 3(a) and 3(b)).

**Figure 4:**
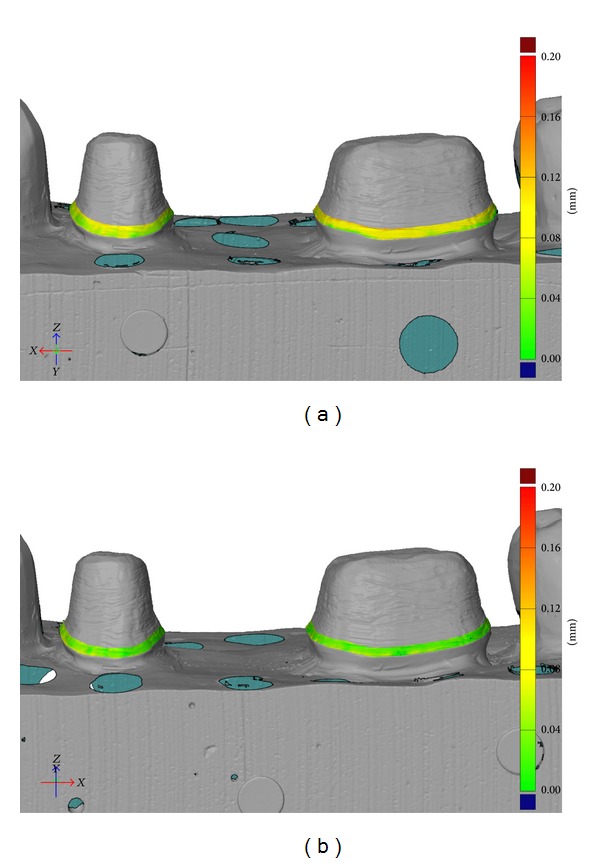
(a) Analysis of the cervical area fit of FDPs produced with the conventional impression technique. The distance from abutment to inside of FDP is represented by color. Green = 0–40 *μ*m and yellow = 70–110 *μ*m. (b) Analysis of the cervical area fit of FDPs produced with the digital impression technique. The distance from abutment to inside of FDP is represented by color. Green = 0–40 *μ*m and yellow = 70–110 *μ*m.

The mean cervical area discrepancy was small for both techniques: 44 *μ*m for the digital impression technique and 69 *μ*m for the conventional impression technique; *P* < 0.001 (Table 3, Figures 4(a) and 4(b)).

## 4. Discussion

The purpose of this study was to evaluate the marginal and internal fit of CNC-milled CoCr 3-unit FDPs produced from digital and conventional impressions using a triple-scan protocol. The results show that the digital impression technique produced FDPs with a more precise mean internal and cervical fit as compared to the conventional impression technique. The CAD software was preset with a cement spacer gap of 30 *μ*m for the cervical area (the 0.5 mm closest to the finishing line) and 60 *μ*m for axial and occlusal areas. In the present study, the cervical area discrepancy was 44 *μ*m and 69 *μ*m, respectively, for the digital and conventional impression techniques, which is reasonably close to the preset gap of 30 *μ*m for the digital technique. The digital impression technique could thereby be regarded as more accurate in the critical cervical area. The mean internal discrepancy between abutment and retainer was 93 *μ*m and 117 *μ*m, respectively, for the digital and conventional impression techniques, indicating that the mean internal discrepancy tends to exceed the preset spacer, especially in the occlusal area. However, both impression techniques resulted in an acceptable fit on the axial walls but an increased discrepancy in the occlusal area, especially for the conventional impression technique. The conventional technique also resulted in overall greater variability compared to the digital impression technique.

Earlier studies have reported larger discrepancies in the occlusal area [10, 13, 23], and this is supported by the results in the present study where FDPs produced from the conventional impression technique showed a discrepancy ranging from 160 *μ*m to 400 *μ*m and the digital technique showed discrepancies from 80 *μ*m to 400 *μ*m. However, the maximum distances obtained with this analysis could be misleading, due to defects in the point cloud that in turn lead to small holes in the CAD file. These defects may occur when scanning the inside of the FDP, because of the triangulation technique. When measuring distances, a hole in the CAD file results in a maximum distance of 400 *μ*m. Thus, a maximum discrepancy of 400 *μ*m may be due to a discrepancy of 400 *μ*m, or to a hole in the point cloud. However, since these holes are very small they do not affect the overall mean distance calculated from 250.000 to 400.000 points. Overall, the mean internal discrepancy for the premolar was slightly smaller than that for the molar with both techniques. A larger occlusal area on the molar might explain this since the occlusal area had greater discrepancies compared to the axial area. When looking at the absolute marginal gap, there was no statistically significant difference in mean value. The results for absolute marginal gap are comparable to earlier studies on milled CoCr and Zirconia, 185–260 *μ*m [3] and 94–181 *μ*m [24]. The marginal gap measured with this method consists of marginal gap and over- or underextension of the FDP margins. Thus, the results should only be compared to other studies on absolute marginal gap. Also, the triple scan protocol using the ATOS scanner may not be the most suitable tool for measuring the absolute marginal gap, due to a problem in capturing the outermost thin margin of the FDP. This results in a CAD margin that fails to mirror the physical margin, and therefore the measured gap is larger.

Marginal gap may be easier to measure by using a micro-CT technology technique as reported by Borba et al., using cross-sectional images [25]. With this technique the marginal gap can easily be defined and measured, although measurements will only be carried out in the defined slices. Prasad and Al-Keraif used spiral scan microtomography to make a 3D measurement of the spatial gap values in the cervical area and a traveling microscope to measure marginal gap at 16 equidistant points [26]. Rungruanganunt et al. used micro-CT technology for 3D evaluation of precementation space by imaging ultralight body VPS impressions of the precementation space; however, with this technique marginal fit was not measured [27]. One could speculate if the coordinate measuring machine (CMM) technique, often used for fit assessment of implant-supported frameworks, would be better at capturing the marginal gap. However, to the authors knowledge CMM has not been used for evaluation of fit of tooth supported FDPs.

All of the FDPs produced with the digital impression technique were easily seated on their corresponding study cast. In contrast, one of the FDPs produced with the conventional impression technique could not be seated on the study cast. This was probably due to an inaccurate impression and/or fabrication of the working cast, since the FDP was easily seated on the working cast.

In the present study, stone casts were used as study casts which could be a confounder; however, no dimensional changes were seen before and after conventional impression of the stone cast and there were no problems in reproducing the finishing line. In the laboratory setup, no saliva was present giving optimal conditions for impression taking with both techniques. Whether this favors any of the techniques cannot be established from the present study. Nevertheless, Flügge and colleagues reported that the iTero intraoral scanner produces more precise impressions from stone casts than from patients [28].

The results of the present study showed that the digital impression technique was more reliable and generated 3-unit FDPs with a significantly closer fit compared to the VPS technique in a laboratory setup. However, further clinical studies are needed to confirm that comparable results can be achieved in vivo on small and large-span FDPs.

Within the limitations of this study, it can be concluded that in a laboratory test situation the digital impression technique was more precise than conventional impressions using VPS impression material. The fit of the FDPs was good and with a cervical area discrepancy of 44 *μ*m for the digital and 69 *μ*m for the conventional technique they would all be clinically acceptable. The triple-scan protocol for 3D fit assessment can be used to measure the accuracy of fit of tooth supported FDPs as it provides a good 3D measurement of all surfaces with the exception of assessing the marginal gap.

The null hypothesis is therefore rejected and the alternative hypothesis is accepted, since the digital impression technique produced FDPs with a significantly closer fit.

## Figures and Tables

**Table 1:** Mean internal discrepancy in *μ*m and standard deviation (SD) between abutment surface and inside surface of FDP produced with conventional and digital impression techniques.

Impression technique	*n*	Internal discrepancy to master model (*μ*m)					
		Premolar 34		Molar 36		FDP 34–36	
		Mean	(SD)	Mean	(SD)	Mean	(SD)
Conventional	10	100	6.7	127	15.7	117	11.6
Digital	10	91	8.8	95	8.5	93	8.2
Significance (2-tailed) (*P* < 0.01)		0.016		<0.001		<0.001	

**Table 2:** Absolute marginal gap between finish line and crown margin for conventional and digital impression techniques.

Impression technique	*n*	Absolute marginal gap (*μ*m)					
		Premolar 34		Molar 36		FDP 34–36	
		Mean	(SD)	Mean	(SD)	Mean	(SD)
Conventional	10	140	30.9	154	19.0	147	22.6
Digital	10	146	44.5	139	24.7	142	32.6
Significance (2-tailed) (*P* < 0.01)		0.788		0.100		0.425	

**Table 3:** Cervical area discrepancy for conventional and digital impression techniques.

Impression technique	*n*	Cervical area discrepancy (*μ*m)					
		Premolar 34		Molar 36		FDP 34–36	
		Mean	(SD)	Mean	(SD)	Mean	(SD)
Conventional	10	61	9.1	77	19.3	69	12.4
Digital	10	44	11.7	44	6.4	44	8.2
Significance (2-tailed) (*P* < 0.01)		0.003		<0.001		0.001	
